# Kinin release from human kininogen by 10 aspartic proteases produced by pathogenic yeast *Candida albicans*

**DOI:** 10.1186/s12866-015-0394-8

**Published:** 2015-03-04

**Authors:** Andrzej Kozik, Mariusz Gogol, Oliwia Bochenska, Justyna Karkowska-Kuleta, Natalia Wolak, Wojciech Kamysz, Wataru Aoki, Mitsuyoshi Ueda, Alexander Faussner, Maria Rapala-Kozik

**Affiliations:** Faculty of Biochemistry, Biophysics, and Biotechnology, Jagiellonian University in Krakow, Gronostajowa 7, 30-387 Krakow, Poland; Faculty of Pharmacy, Medical University of Gdansk, Al. Hallera 107, 80-416 Gdansk, Poland; LipoPharm.pl, Koscielna 16A, 83-210 Zblewo, Poland; Department of Applied Physics, Graduate School of Engineering, Osaka University, 2-1 Yamadaoka, Suita, Osaka, 565-0871 Japan; Division of Applied Life Sciences, Graduate School of Agriculture, Kyoto University, Sakyo-ku, Kyoto, 606-8502 Japan; Institut für Prophylaxe und Epidemiologie der Kreislaufkrankheiten, Ludwig-Maximilians-University, Pettenkoferstrasse 9b, 80336 Munich, Germany

**Keywords:** Candidiasis, Human kininogen, Met-Lys-bradykinin, Des-Arg-kinins, Bradykinin B2-subtype receptors, *Pichia pastoris*

## Abstract

**Background:**

*Candida albicans* yeast produces 10 distinct secreted aspartic proteases (Saps), which are some of the most important virulence factors of this pathogenic fungus. One of the suggested roles of Saps is their deregulating effect on various proteolytic cascades that constitute the major homeostatic systems in human hosts, including blood coagulation, fibrinolysis, and kallikrein-kinin systems. This study compared the characteristics of the action of all 10 Saps on human kininogens, which results in generating proinflammatory bradykinin-related peptides (kinins).

**Results:**

Recombinant forms of Saps, heterologously overexpressed in *Pichia pastoris* were applied. Except for Sap7 and Sap10, all Saps effectively cleaved the kininogens, with the highest hydrolytic activity toward the low-molecular-mass form (LK). Sap1–6 and 8 produced a biologically active kinin—Met-Lys-bradykinin—and Sap3 was exceptional in terms of the kinin-releasing yield (>60% LK at pH 5.0 after 24 hours). Des-Arg^1^-bradykinin was released from LK by Sap9 at a comparably high yield, but this peptide was assumed to be biologically inactive because it was unable to interact with cellular B2-type kinin receptors. However, the collaborative actions of Sap9 and Sap1, −2, −4–6, and −8 on LK rerouted kininogen cleavage toward the high-yield release of the biologically active Met-Lys-bradykinin.

**Conclusions:**

Our present results, together with the available data on the expression of individual *SAP* genes in candidal infection models, suggest a biological potential of Saps to produce kinins at the infection foci. The kinin release during candidiasis can involve predominant and complementary contributions of two different Sap3- and Sap9-dependent mechanisms.

## Background

The secretion of active proteases is one of the most successful strategies used by microbial pathogens to colonize and infect human hosts [1]. By hydrolyzing proteinaceous targets in the host organism, including the proteins in cell membranes and extracellular matrix, these enzymes allow the pathogen to penetrate tissues and acquire nutrients. These enzymes also play important roles in evading the immune system by cleaving immune regulatory proteins [2], or deregulating the major homeostatic systems of the host that require cascade-activated proteolysis such as the blood coagulation [3], fibrinolysis [4], complement [5], and kallikrein-kinin system [6].

*Candida albicans* is one of the most common fungal pathogens in humans [7]. This yeast-like fungus can release as many as 10 aspartic class proteases into the extracellular space [8]. *C. albicans* is part of the physiologic human microbiota, but, under some circumstances that are generally related to immune system weaknesses in the host, it can convert to a dangerous pathogen that causes diseases of variable severity. These candidiases can range from relatively mild and easily curable superficial infections of the skin and mucous membranes to life-threatening deep-seated invasions in the inner organs, fungemia, and systemic diseases with high mortality rates [9]. It is assumed that the number and diversity of secreted aspartic proteases (Saps) from *C. albicans* are needed to successfully colonize the variety of niches present in humans [10], but this hypothesis is still unsatisfactorily evidenced by experimental data. In particular, relatively few studies are devoted to systematically comparing the actions of all 10 Saps on a single proteinaceous substrate.

Bradykinin-related peptides, which are collectively called kinins, are proteolytically released from the serum proteins, kininogens [11-13]. Serine proteases, called kallikreins are primarily devoted to this task [14], but during pathological states, including those associated with microbial infections, other proteases, either from the host or the pathogen, can supplement the actions of the kallikreins [15-18]. Due to the multiple functions of kinins required to regulate various physiological processes, as well as their participation in almost every inflammatory state [11,12,19], the kallikrein-kinin system is considered a major system required for biochemical homeostasis in humans. Overactivated kinin production reportedly occurs in infections caused by numerous bacterial species [6,20]. Regarding candidal infections, Kaminishi et al. [21] was the first to report that a purified major extracellular protease of *C. albicans* possesses kinin-releasing potential, albeit indirectly, based on the activation of an upstream-acting zymogen in the kinin-generating cascade (factor XII). The direct release of kinins from kininogens was later confirmed for mixtures of proteases that were released into the culture medium by several *Candida* species [22] and the purified *C. albicans* protease, which was unequivocally identified as Sap2 [23]. Sap2 is believed to be the predominant protease secreted by *C. albicans* when cultured in protein-rich media, but the *SAP* gene expression profile depends on the fungal morphology, and the high expression of different *SAP* genes has been reported in various infection models [24,25].

This study compares the characteristics of kininogen cleavage and concomitant kinin release due to all 10 individual Saps, used in recombinant purified forms. Here, we report the exceptionally high kinin-forming activity of Sap3. Moreover, the active kinins are produced with a high yield by mixtures of Sap9 and several other Saps.

## Results

**Figure 1:**
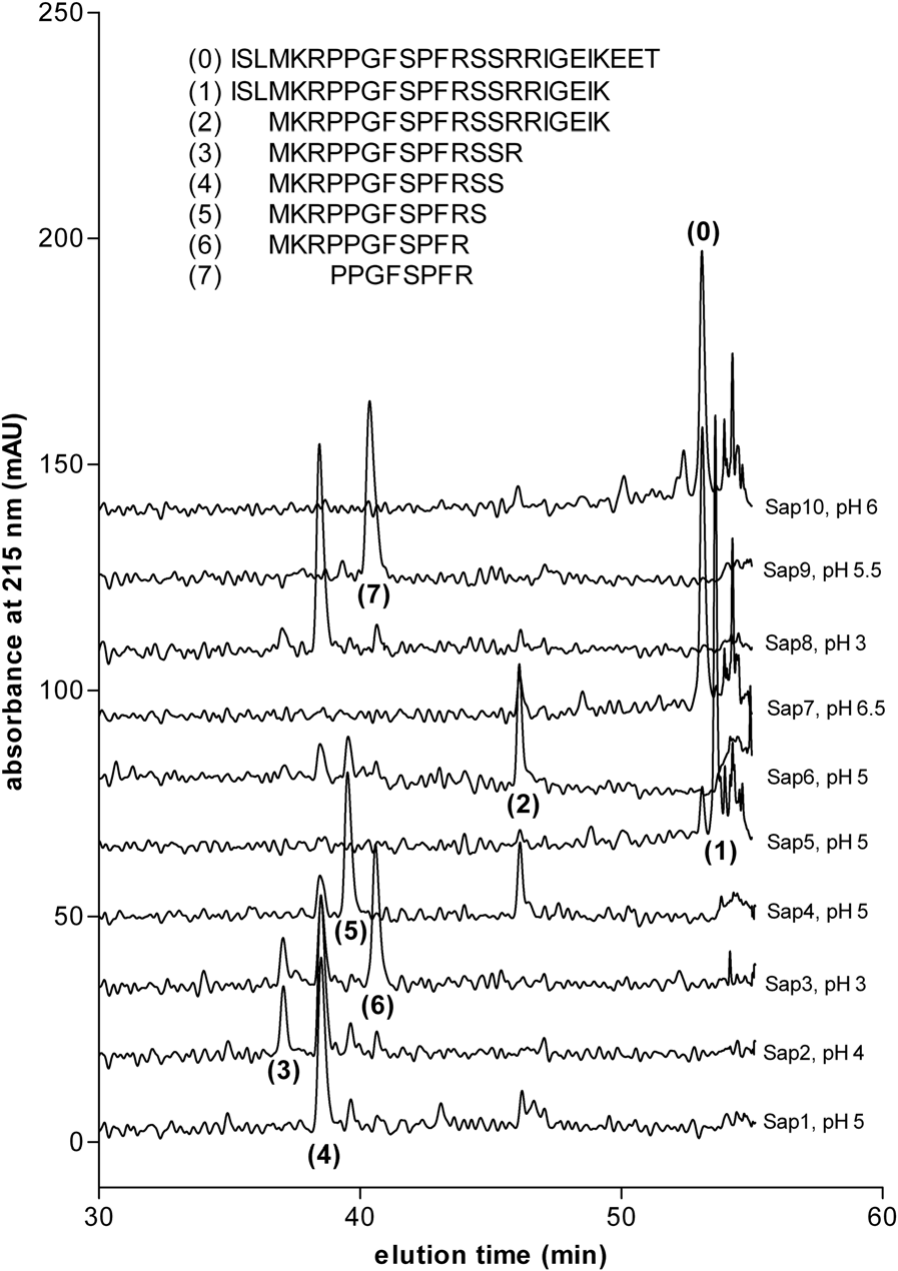
**HPLC/MS characteristics of the Sap-catalyzed cleavage of the HK-D4 peptide.** Ten μM of the synthetic peptide HK-D4 (which has the ISLMKRPPGFSPFRSSRIGEIKEET amino acid sequence) were treated with recombinant Sap1–10 in citrate (50 mM) or phosphate buffers (25 mM) at the optimal pH for the general proteolytic activity [26] of each individual Sap (specified in the figure) at an enzyme:substrate molar ratio of 1:50 for 24 hours at 37°C. The reaction was stopped using HCl, and the samples were analyzed using reversed-phase HPLC on an Eurosil Bioselect 300–5 C-18 column (Knauer) in a TFA-water-ACN binary gradient system. The fractions, which were collected at the major absorbance peaks (215 nm), were evaporated and analyzed using ESI-MS/MS in order to determine their amino acid sequence.

The mechanism responsible for the Sap-catalyzed cleavage of domain 4 of human kininogen was studied using a synthetic peptide—denoted as HK-D4—because its amino acid sequence (ISLMKRPPGFSPFRSSRIGEIKEET) can be obtained from the kinin-containing region of the kininogen molecule. The cleaved products were separated using high performance liquid chromatography (HPLC), and the major peaks were collected and analyzed to determine their sequences using tandem mass spectrometry (MS/MS). Comparative chromatograms of the samples, obtained after incubating HK-D4 with all individual recombinant Saps for long periods of time (i.e., 24 hours) at the optimal pH for general proteolytic activity (against standard proteinaceous substrates such as casein) [26] are shown in Figure 1. HK-D4 was effectively cleaved by all Saps, except Sap7 and Sap10. The qualitative distributions of the major formed products are similar for Sap1–6 and 8; however, Sap3 demonstrated the ability to release a kinin-like peptide—Met-Lys-bradykinin (MKRPPGFSPFR)—at the highest yield (approximately 50%). Moderate-to-small amounts of the same peptide were also detected among the minor products of HK-D4 that were cleaved by Sap2, which agrees with our recent characterization of the kininogenase activity of the natural, purified Sap2 [23] and Sap1, Sap4, and Sap8. In contrast, Sap9 showed a higher preference to hydrolyze the peptide bonds at the carboxyl side of the arginine residues than Sap3, and thus could easily cleave the bond between the arginine and proline located at the N-terminal side of the internal bradykinin sequence. Due to this exceptional specificity, the HK-D4 cleavage pattern caused by Sap9 was unique, and a different, kinin-related peptide—des-Arg^1^-bradykinin (PPGFSPFR)—was formed at a high yield (approximately 80%).

**Figure 2:**
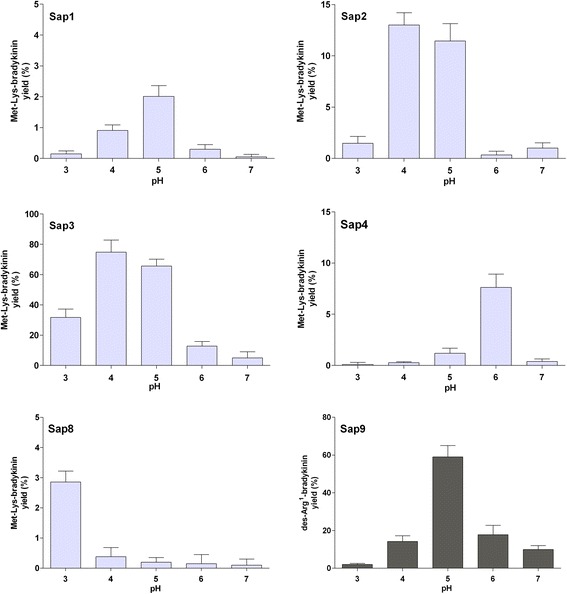
**Effect of pH on Sap-catalyzed kinin release from the HK-D4 peptide.** HK-D4 (10 μM) was cleaved using 0.2 μM Saps (1–4, 8, and 9) in 50 mM citrate buffers (pH 3.0–5.0) or 25 mM phosphate buffers (pH 6.0–7.0) for 24 hours at 37°C. The reaction was stopped using HCl (0.33 M), and the samples were analyzed using HPLC as specified in Figure 1. The amounts of Met-Lys-bradykinin or des-Arg^1^-bradykinin (for Sap9) that formed were estimated based on the peak areas and are expressed relative to the maximum possible amount (calculated using the molarity of the reaction substrate). Data represent mean values from the analysis of three independent samples (three separate digests) ± the standard deviation.

The dependence of the kinin yield on the pH for all kinin-forming Saps (i.e., Sap1-4 and 8–9, see Figure 1) is illustrated in Figure 2. Only Sap3 presented the optimal kinin-forming activity at pH markedly shifted from the optimum with the general protease substrates (pH 3 in this case) towards a more neutral pH. Thus, > 70% of HK-D4 was cleaved by Sap3 towards Met-Lys-bradykinin production at pH 5. Importantly, kinin release was still detectable at ≥ pH 6.

**Figure 3:**
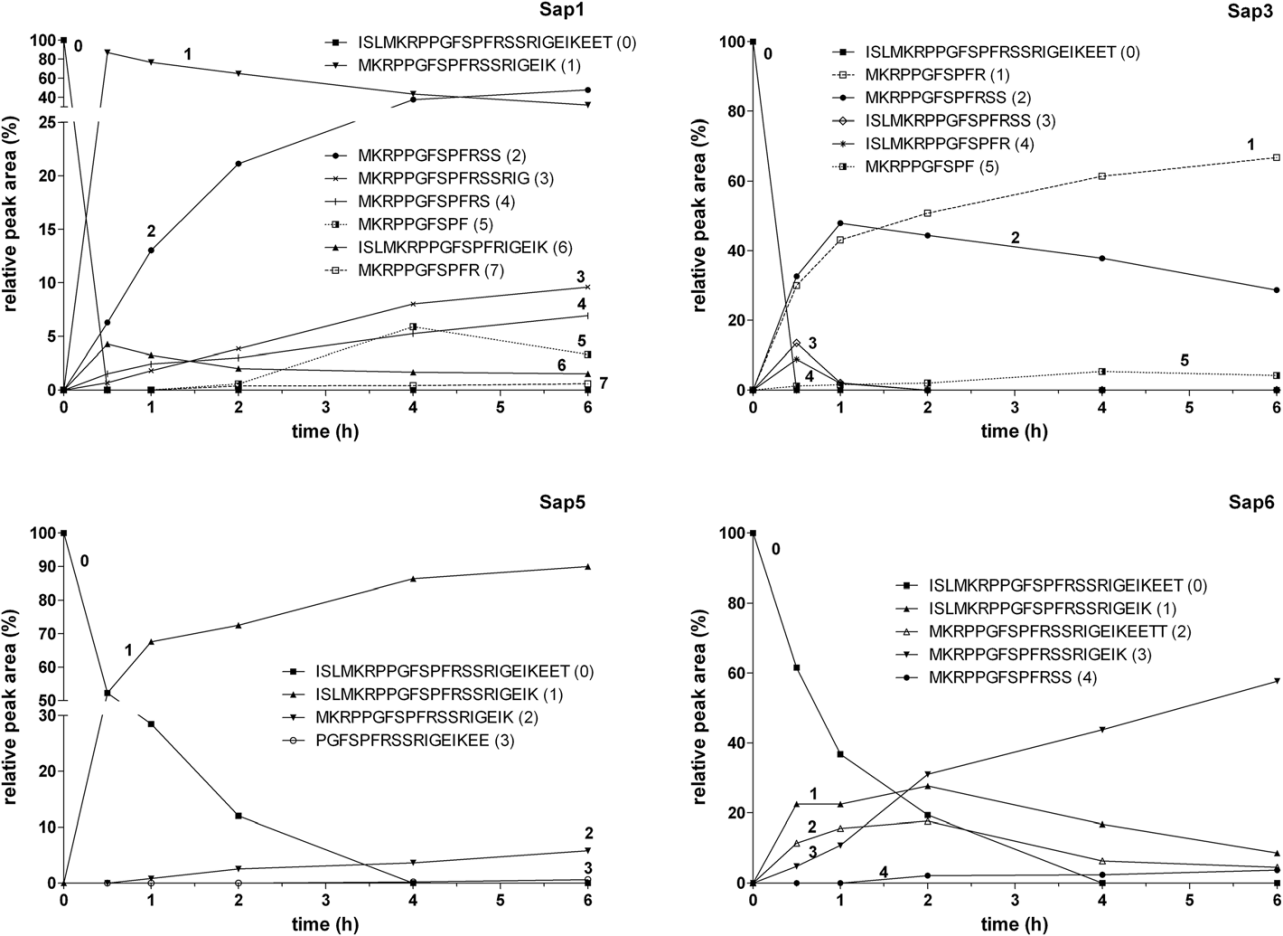
**Time course of HK-D4 cleavage by Sap1, Sap3, Sap5, and Sap6.** HK-D4 (10 μM) was cleaved using 0.2 μM Sap in 50 mM citrate buffer (pH 5.0) at 37°C for the specified time. The reaction was stopped using HCl, and the samples were analyzed using HPLC as specified in Figure 1. The results from representative kinetic experiments are shown. The areas under the peaks of the early, major cleavage products, as well as the kinin-related peptides, are expressed relative to the substrate at the beginning of the reaction.

The time course of HK-D4 cleavage by the Saps reveals the order by which particular peptide bonds are preferably cleaved (Figure 3). The cleavage preferences of Sap1 (Figure 3), Sap2, Sap4, and Sap8 (data not shown) are essentially the same as that previously reported for natural, purified Sap2 [23]. The bonds after Lys^22^ and Leu^3^ were hydrolyzed at the highest rates. Thus, the N-terminus of the kinin to be formed (Met-Lys-bradykinin) was established during the very early stages of Sap-catalyzed HK-D4 hydrolysis (within the first hour) and persisted on a longer time scale. A number of additional cleavages occurred downstream from the kinin C-terminus, which ended up with the exposition of Ser^16^, and usually Ser^15^ although at a slower rate. Thus, the MKRPPGFSPFRSS and MKRPPGFSPFRS peptides could be considered the major final products of HK-D4 cleavage following treatment with this set of Saps. Hydrolyzing the bond after Arg^14^, which completes the formation of Met-Lys-bradykinin, occurred at very slow rates when treated with Sap2, −4, −1, and −8 (in that order in terms of kinin yield). Due to the high preference of Sap3 for hydrolyzing the bonds after Arg^14^ and Arg^17^ (Figure 3), Met-Lys-bradykinin was one of the major end products of HK-D4 cleavage, together with the MKRPPGFSPFRSS and MKRPPGFSPFRSSR peptides.

The cleavage of HK-D4 by Sap5 and Sap6 (Figure 3) was limited to the hydrolysis of the two bonds after Lys^22^ and Leu^3^. Sap5 quickly cleaved the bond after Lys^22^ to form the ISLMKRPPGFSPFRSSRIGEIK peptide, at the expense of which the MKRPPGFSPFRSSRIGEIK peptide appeared after a longer period of time at a much lower rate. These two cleavages alternatively occurred at high and comparable rates due to the action of Sap6, which lead to the fast formation of the ISLMKRPPGFSPFRSSRIGEIK and MKRPPGFSPFRSSRIGEIKEET peptides that thereafter were quickly converted to the final major product, MKRPPGFSPFRSSRIGEIK. Among other very minor products that resulted from the actions of Sap5 and Sap6 on HK-D4, no kinin-like peptides were found using the applied chromatographic method (although they could be visualized using MS; see below). The unique time course of HK-D4 cleavage by Sap9 (data not shown) was characterized by the very fast formation of des-Arg^1^-bradykinin, which was only preceded by quickly disappearing precursors such as the ISLMKRPPGFSPFR or PPGFSPFRSSRIG peptides.

**Figure 4:**
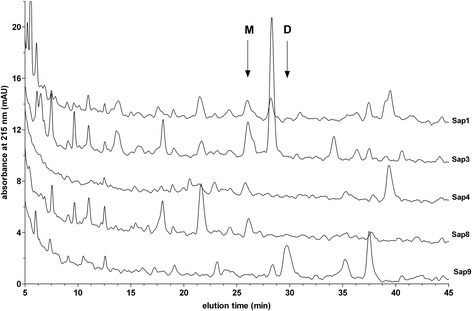
**HPLC profiles of the fragmentation of LK by selected Saps.** LK samples (1.5 μM) were digested with 0.03 μM of Sap1, −3, −4, −8, and −9 in the citrate buffer (pH 5.0) at 37°C for 24 hours. The reaction was stopped using pepstatin A (10 μM), followed by acidification with HCl (0.33 M). The samples were analyzed using HPLC on the Luna C18(2) 5 μm 4.6 × 250 mm column (Phenomenex) in a TFA water-ACN binary gradient system, as described in the Materials and Methods section. Arrows indicate the retention times of the Met-Lys-bradykinin (M) and des-Arg^1^-bradykinin (D) standards.

An analysis using sodium-dodecyl-sulphate polyacrylamide-gel electrophoresis (SDS-PAGE) showed that Sap1–6, −8, and −9 cleaved native human kininogens, with a high preference for the low-molecular-mass form (LK) in comparison with the high-molecular-mass form (HK) (data not shown). Using the optimized HPLC method, the clearly distinguishable peak of Met-Lys-bradykinin was visible on the chromatograms obtained after LK digestion by Sap1–4 and −8, while Sap9 resulted in the intense peak of des-Arg^1^-bradykinin (Figure 4). To identify all kinin-like peptides formed by all individual Saps (even those formed in very small amounts), the more sensitive liquid chromatography-coupled tandem mass spectrometry (LC-MS/MS) method was applied. We found that (1) in addition to Sap1–4 and −8, Sap5 and Sap6 also exhibited kinin-forming activities toward the LK substrate and (2) each individual Sap produced multiple kinin-like peptides (Table 1). These included, in addition to the predominant Met-Lys-bradykinin (or des-Arg^1^-bradykinin for Sap9), bradykinin itself, and (1) occasional traces of kinin derivatives without the C-terminal Arg residues (Sap1, Sap2, Sap4, Sap8, Sap9), (2) Leu-Met-Lys-bradykinin (Sap2, Sap3, Sap9), and (3) versions of kinins with hydroxyproline (POH) at the third position of the bradykinin sequence, as could have been expected from native human kininogens [27], oxidized Met residue (MO), or both (all Saps except Sap5).

**Table 1:** **Kinin-like peptides in the Sap-treated LK samples that were identified using LC-MS/MS**

**Sap**	**Kinin-like peptide**	**Molecular mass (Da)**
Sap1	**MKRPPGFSPFR**	1318.8
	MKRP(POH)GFSPFR	1334.7
	(MO)KRPPGFSPFR	1334.7
	MKRPPGFSPF	1162.8
	RPPGFSPFR	1059.8
	PPGFSPFR	903.4
Sap2	**MKRPPGFSPFR**	1318.8
	MKRP(POH)GFSPFR	1334.7
	(MO)KRPPGFSPFR	1334.7
	RPPGFSPFR	1059.8
	LMKRPPGFSPFR	1431.9
	MKRPPGFSPF	1162.8
	RP(POH)GFSPF	919.7
Sap3	**MKRPPGFSPFR**	1318.7
	**MKRP(POH)GFSPFR**	1334.7
	(MO)KRPPGFSPFR	1334.7
	(MO)KRP(POH)PGFSPFR	1350.7
	RPPGFSPFR	1059.8
	**LMKRPPGFSPFR**	1432.0
	LMKRP(POH)GFSPFR	1447.8
	L(MO)KRPGFSPFR	1447.8
Sap4	**MKRPPGFSPFR**	1318.7
	MKRP(POH)GFSPFR	1334.7
	(MO)KRPPGFSPFR	1334.7
	(MO)KRP(POH)PGFSPFR	1350.7
	RPPGFSPFR	1059.8
	RP(POH)GFSPF	919.7
Sap5	MKRPPGFSPFR	1318.8
	RPPGFSPFR	1059.8
Sap6	MKRPPGFSPFR	1318.8
	RPPGFSPFR	1059.8
	MKRP(POH)GFSPFR	1334.7
Sap8	**MKRPPGFSPFR**	1318.7
	MKRP(POH)GFSPFR	1334.7
	(MO)KRPPGFSPFR	1334.7
	(MO)KRP(POH)PGFSPFR	1350.7
	RPPGFSPFR	1059.8
	MKRPPGFSPF	1162.8
Sap9	MKRPPGFSPFR	1318.9
	RPPGFSPFR	1059.6
	**PPGFSPFR**	903.5
	**P(POH)GFSPFR**	919.5
	LMKRPPGFSPFR	1432.0
	LMKRP(POH)GFSPFR	1447.8
	PPGFSPF	747.4

LK samples (1.5 μM) were digested with 0.03 μM Sap in citrate buffer (pH 5.0) at 37°C for 24 hours. The reaction was stopped using pepstatin A (at a final concentration of 10 μM), and the samples were acidified with HCl (0.33 M) and subjected to LC-MS/MS analysis using the Bruker HCTultra ETDII IT mass spectrometer that was equipped with an ESI ion source and ETD II fragmentation module and was coupled to the Dionex Ultimate 3000 UHPLC system. MS/MS data were analyzed using in-house Mascot server (version 2.3.0), and the peptides were identified by searching the Swiss-Prot database. The peptides marked in **bold** generated peaks with the highest intensities (> 10^5^ arbitrary units), which exceeded by ≥ 1 order of magnitude in comparison with the other peptides.

**Figure 5:**
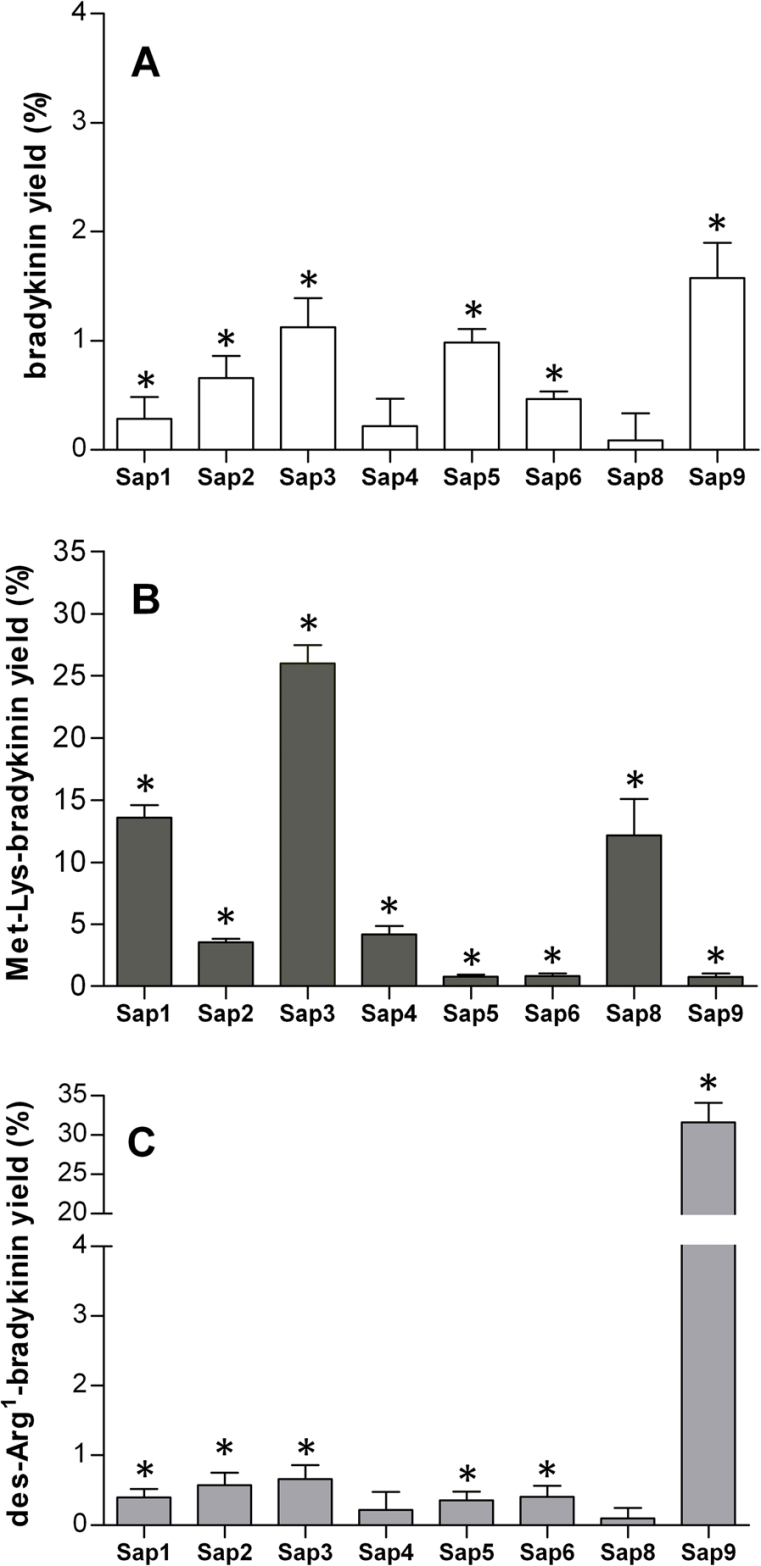
**Distribution of bradykinin, Met-Lys-bradykinin, and des-Arg** ^**1**^ **-bradykinin in the Sap-digested LK samples.** LK samples (1.5 μM) were digested using 0.03 μM Sap in the citrate buffer (pH 5.0) at 37°C for 6 hours. After sequentially stopping the reaction using pepstatin A and HCl, the obtained peptide mixtures were separated on the Luna C18 column in a TFA water-ACN binary gradient system. The fractions were collected at the retention times that correspond to the bradykinin, Met-Lys-bradykinin, and des-Arg^1^-bradykinin standards, evaporated to dryness, and redissolved in the assay buffer of the ELISA kit in order to quantitatively determine the kinin concentrations. The corresponding fractions, obtained from the HPLC separation of intact (undigested) LK served as the controls, and the kinin concentrations, determined in these fractions are subtracted from those in the Sap-digested LK samples. The corrected amount of each of the three kinins is expressed relative to the maximum possible amount of all kinins (as calculated using the molarity of LK in the sample). Results, obtained from two independent experiments (two LK-digests independently analyzed by HPLC for each Sap), with three replicate ELISA measurements (in three different wells) for each fraction obtained during each HPLC separation, are presented as the mean values ± standard deviation. Asterisks denote the statistical significance of the difference between the kinin levels in the Sap-treated and undigested LK samples (t-Student test, p < 0.05). The data plotted in the three panels are for bradykinin **(A)**, Met-Lys-bradykinin **(B)**, and des-Arg^1^-bradykinin **(C)**.

The relative distributions of the main kinins were quantitatively determined in the Sap-treated LK samples using the HPLC analysis, collecting fractions at the retention times for bradykinin, Met-Lys-bradykinin, and des-Arg^1^-bradykinin, and enzyme-linked immunosorbent assay (ELISA) for kinins in these fractions (Figure 5). While detectable, small amounts of bradykinin were released from LK by all studied Saps, Sap9 produced this “main” kinin at the relatively highest yield, which approached 2% of the maximum releasable kinin content in the sample. All tested Saps produced Met-Lys-bradykinin at the yield, which decreased in the order of Sap3 > > Sap1 ≈ Sap8 > Sap2 ≈ Sap4 > > Sap5 ≈ Sap6 ≈ Sap9; however, no Sap could favorably compete with Sap3 over the long term (i.e., 24 hours) in terms of the kinin release yield (>60% LK). At a comparably high yield, Sap9 could produce des-Arg^1^-bradykinin that would barely be detectable in the LK digests obtained after long incubation with other Saps.

**Figure 6:**
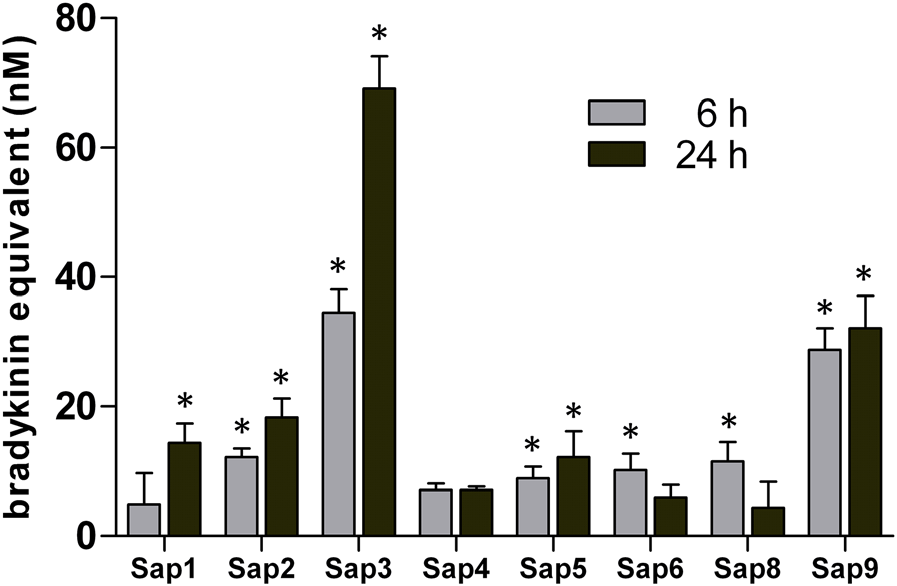
**The amounts of the B2 receptor-interacting peptides in the Sap-digested LK samples.** LK samples (1.5 μM) were digested with 0.03 μM Sap in the citrate buffer (pH 5.0) at 37°C for 6 or 24 hours, the reaction was stopped using pepstatin A (at a final concentration of 10 μM), and the samples were analyzed for kinin content using a competitive radioreceptor assay that used B2 receptor-overexpressing HEK293 cells. The calibration plot for the assay was prepared using a bradykinin standard. The results are corrected by subtracting the values, determined in the undigested LK sample. Data represent mean values from three separate radioreceptor binding analyses (three independently prepared cultures of B2 receptor-bearing cells), with the measurements performed in triplicates within each experiment. The error bars represent the standard deviations; asterisks denote the statistical significance of the differences between Sap-treated and undigested LK samples (t-Student test, p < 0.05).

The Sap-treated LK samples were additionally analyzed for their interactions with HEK293 cells, which were engineered to overexpress B2-subtype kinin receptors on the surface (Figure 6). This part of our study aimed to assess putative biological activity, typical of kinins, in the peptide mixtures generated by Saps from human kininogen. Using this type of ligand-receptor radioassay [28], the highest amount of “bradykinin equivalents” was found in the LK digests obtained using Sap3, followed by those generated by Sap9. Interpreting these results was not straightforward because attempts to calibrate the radioassay with synthetic des-Arg^1^-bradykinin failed to detect any interactions between this peptide and the B2 receptors (results not shown), contrary to Met-Lys-bradykinin which binds to receptors although at a 10-fold lower affinity than bradykinin [23,29]. Thus, the peak of B2 receptor-binding activity, which was found in the Sap9-treated LK samples (Figure 6), was probably due to the bradykinin that developed in the largest amount in comparison with all other Saps (Figure 5). The highest peak recorded for Sap3 was obviously due to the large amount of the true B2-receptor agonist (Met-Lys-bradykinin) produced from LK by this protease at a nearly stoichiometric yield (Figure 5). Actually, the apparent amount of kinin in this sample was underestimated by the radioassay, which had been calibrated to the bradykinin standard.

**Figure 7:**
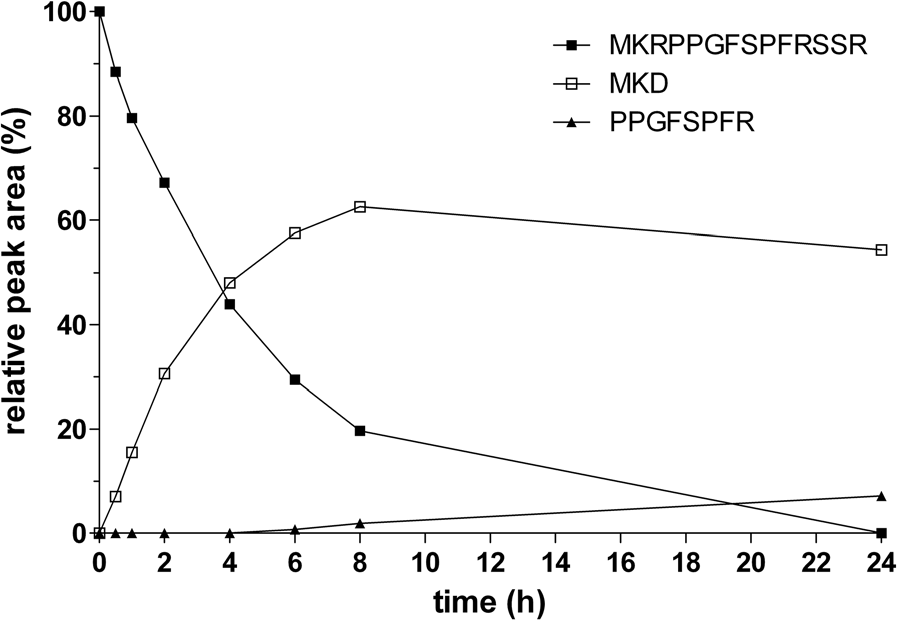
**Time course of the cleavage of the MKRPPGFSPFRSSR peptide using Sap9.** MKRPPGFSPFRSSR peptide (5 μM) was cleaved using 0.1 μM Sap9 in 50 mM citrate buffer (pH 5.0) at 37°C for the specified time. The reaction was stopped using HCl, and the sample was analyzed using HPLC as specified in Figure 1. The results from a representative kinetic experiment are shown. The areas under the peaks of separated peptides are expressed relative to the substrate at the beginning of the reaction.

The short peptide—des-Arg^1^-bradykinin (PPGFSPFR)—was assumed to be biologically inactive because of its inability to interact with B2 receptors. Therefore, we were very interested to determine if Sap9 can hydrolyze the peptide bond after the third arginine residue in (1) Met-Lys-bradykinin, thereby inactivating this biologically active kinin formed from the kininogens by the other Saps, and (2) other peptides with an N-terminal Met residue, such as MKRPPGFSPFRSSRIGEIK, MKRPPGFSPFRSS, MKRPPGF-SPFRS, and MKRPPGFSPFRSSR—which are the fast-appearing major products of kininogen cleavage by other Saps—thereby preventing the subsequent conversion to active kinin. Surprisingly, as shown in Figure 7, Sap9 was essentially unable to process these substrates at the N-terminus, suggesting that this protease requires a long stretch of amino acids upstream from the N-terminal side of the internal bradykinin sequence (≥6 residues) to effectively cleave the N-terminal bond between the Arg and Pro residues. However, the C-terminal arginine residue of the kinin sequence—which is absolutely required for interactions between the free kinins and B2 receptors—was still quickly exposed by Sap9. Thus, the Sap-catalyzed cleavage of all substrates with N-terminal methionine converged to a single product, Met-Lys-bradykinin, (i.e., an active kinin).

**Figure 8:**
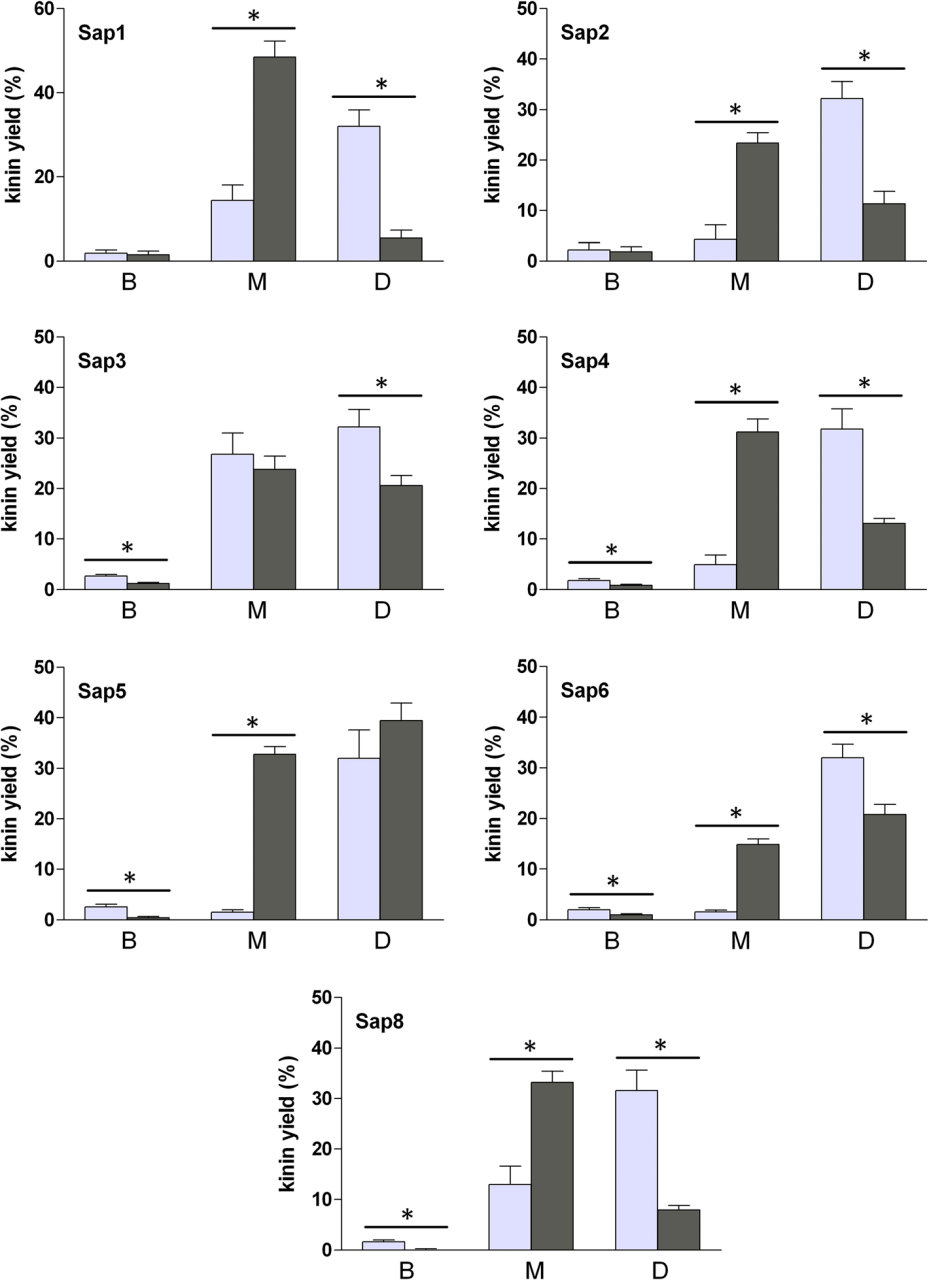
**Comparisons of the generation of bradykinin, Met-Lys-bradykinin, and des-Arg** ^**1**^ **-bradykinin in LK samples that were digested using a mixture of Sap9 and other Saps vs digestion with single Saps.** LK samples (1.5 μM) were digested with either 0.03 μM individual Saps or mixtures of 0.03 μM Sap9 with equimolar amounts of other Saps in the citrate buffer (pH 5.0) at 37°C for 6 hours. After sequentially stopping the reaction with pepstatin A and HCl, the obtained peptide mixtures were separated on the Luna C18 column in a TFA water-ACN binary gradient system. Fractions were collected at the retention times that correspond to the bradykinin (B), Met-Lys-bradykinin (M), and des-Arg^1^-bradykinin standards (D), evaporated to dryness, and redissolved in the assay buffer of the ELISA kit in order to quantitatively determine the kinin concentrations. The results are corrected by subtracting the data, obtained for the undigested LK sample as specified in Figure 5. The amount of each of the three kinins is expressed relative to the maximum possible amount of all kinins (calculated using the molarity of LK in the sample). For each Sap, the light gray bar represents the sum of kinin amounts in two independent samples, treated separately with this Sap and Sap9 while the dark gray bar represents the value for sample of LK that was treated with a mixture of this Sap and Sap9. All data bars represent the mean values of six determinations (averaged as specified in Figure 5) ± standard deviation. Asterisks denote the statistical significance (t-Student test, p < 0.05) of the difference between the indicated kinin levels (generated by a mixture of Sap9 and other Sap vs digestion with two Saps separately).

The unique specificity of Sap9, as determined by our analysis of the synthetic peptides, allows mixtures of Sap9 and Sap1, −2, −4–6, and −8 to demonstrate the strikingly enhanced formation of Met-Lys-bradykinin from kininogen in comparison with the individual actions of these proteases (Figure 8). At the same time, the production of the biologically inactive peptide, des-Arg^1^-bradykinin, was largely quenched. This effect was particularly dramatic with Sap5 and Sap6, which individually produced only trace amounts of Met-Lys-bradykinin, but in the presence of Sap9 the kinin yield exceeded even that of the best individual kinin producer, Sap3. In contrast, the release of Met-Lys-bradykinin from Sap3 was not enhanced in the presence of Sap9, apparently because both these proteases cleave the bond after the Arg residue on the C-side of the internal kinin sequence at a comparably high rate. However, the production of des-Arg^1^-bradykinin by the Sap3 and Sap9 mixture was quenched by about half in comparison with the amount released from the kininogen using Sap9 alone.

## Discussion

The success of *C. albicans* as a pathogen is due to its ability to inhabit various niches within the host organism, including the skin, oral and vaginal mucosa, gastrointestinal tract, and, as in systemic infections, all inner organs and the blood [9,30,31]. This yeast-like fungus can adapt to different environments due to the development of an impressive array of virulence factors [32], including yeast-to-hyphae transition, morphologic switches, numerous surface-exposed adhesins, and hydrolytic enzymes such as Saps. The multiplicity of the effects that determine candidal infections makes it difficult to reliably dissect the relative role of a single factor in the colonization and invasion of host tissues. Thus, eliminating a single virulence factor—e.g., gene mutations—often unsatisfactorily reduces the strain pathogenicity because of the compensating effects of other factors [25,33]. Regarding the 10 different Saps of *C. albicans*, functional redundancy must also exist because numerous attempts to assign specific roles to individual Saps have not led to a general consensus [25,34]. Extensive studies on the expression of individual *SAP* genes in various *in vitro* and *in vivo* models of candidal infections report contradictory results [35,36].

Regarding the role in microbial infections, the kinin-forming system can be compared to a double-edged sword [6]. Primarily, the proinflammatory and vasoactive properties of kinins contribute to the refined multifactorial process that provides host defenses against pathogens. For instance, kinins recruit defense cells such as neutrophils and monocytes to the infection foci [37] and activate many cell types in order to release other, even more potent proinflammatory mediators [38]. However, at least one activity of kinins—enhancing vascular permeability—is beneficial to pathogens, helping them acquire the necessary nutrients from serum and disseminate within the host organism [17]. The upregulation of kinin production has been frequently reported in association with bacterial infections [6,20]. Relatively recently, the hijacking of the kinin-forming system of the host was suggested to occur as part of fungal infections such as candidiasis. *In vitro* studies show that, similar to bacterial pathogens, *C. albicans* can mimic two mechanisms of kinin production used by the host to defeat infections, although in an uncontrolled manner. One mechanism depends on the adsorption of HK and the other components of the contact system on the fungal cell wall [39-41], which resembles the contact activation of kinin release on the surface of numerous host cells [42-45]. The second mechanism involves Saps that activate factor XII [21] or directly release kinins from kininogens [22,23].

In the current study, for the first time, we compared the kininogenase activities of all 10 Saps. Eight of these, Sap1–8, are soluble secreted proteins, while Sap9 and Sap10 are bound to the cell wall via a glycosylphosphatidylinositol (GPI) anchor, although some fraction of these proteins can be shed into the extracellular space [8]. We found that all soluble Saps (except Sap7) can release kinins from human kininogens and strongly prefer LK to HK as the substrate. In each case, the same kinin—Met-Lys-bradykinin—was formed almost exclusively, i.e., in excess over the other identified kinin-like peptides, including bradykinin. Despite its relatively low affinity for B2-type kinin receptors [23,29], Met-Lys-bradykinin is equivalent to bradykinin and kallidin (Lys-bradykinin) in terms of biological activity [46], most likely because it can be easily converted to the latter “main” kinins in biological fluids and tissues that are abundant with nonspecific aminopeptidases [29]. The current, unequivocal identification of the chemical nature of the formed kinin peptides—together with the findings that the unfractionated peptide mixtures generated by Sap1-6 and Sap8 from LK interact with B2 receptors (Figure 6)—provides strong support for the hypothesis that these soluble Saps can release biologically active kinins at infection sites, as they are likely to encounter the appropriate amounts of kininogens to be cleaved [47,48]. The qualitatively uniform picture observed for all individual soluble Saps suggests a high degree of redundancy between these Saps with respect to the postulated kinin-forming functions.

Quantitatively, however, the kininogenase activity of Sap3 so strongly exceeded those of the other Saps that it should be considered the predominant player in putative Sap-dependent kinin production at the sites of candidal infections. At sufficient Sap3 levels, Sap1–2, Sap4–6, and Sap8 would only comprise a fungal proteolytic reserve for kininogen cleavage. However, their significance would increase in the rather unlikely case of Sap3 being absent at the infection site, not only due to their ability to generate a small but persistent stream of kinins but also the quick release of peptides with extended C-terminal sequences (in comparison with receptor B2-interacting kinins). These peptides have occasionally been reported to exert biological effects that are typical of kinins in animal models [16], but this is most likely due to additional processing by tissue carboxypeptidases.

Of the two Saps that bind to the fungal cell wall via the GPI anchor, Sap10 was unable to cleave the kininogens at all; in contrast, Sap9 rapidly and nearly completely excised from LK des-Arg^1^-bradykinin (PPGFSPFR) that is assumed to be biologically inactive because it does not interact with B2-type kinin receptors. We also found that its derivative without the C-terminal arginine—i.e., des-Arg^1,9^-bradykinin (PPGFSPF)—that can potentially form in host tissues due to the actions of carboxypeptidases, is not a high-affinity ligand of B1 receptors (data not shown). However, a specific Sap9-dependent inactivation of the kininogens at the fungal cell wall is unlikely in the light of the previous findings that in virtually any infection model in which *SAP9* gene expression is detected, some soluble Saps are also produced [25,49].

The impressive adaptability of *C. albicans* to very different environments within the host organism depends on the compatibility of various virulence factors, such as Saps, with the broad range of environmental parameters. pH is one of the most important parameters to consider [26]. Indeed, the optimal pH for the enzymatic activity of the 10 *C. albicans* Saps ranges between 2–7, which is sufficiently broad to assure that ≥ 1 Sap will effectively hydrolyze proteinaceous targets in each of the major niches that are colonized and infected by this fungus, including the skin (pH 4.7–7), vagina (pH 4), oral cavity (pH 6.3–6.8), blood (pH 7.4), and different parts of the gastrointestinal tract (pH 2–8). Of the two Saps that are hypothesized to play critical roles in kinin generation at the sites of candidal infections, Sap3 optimally acts at an acidic pH. However, this Sap is exceptional among all Saps, not only because of its high kinin-releasing activity against LK, but also because the optimal pH for this process markedly shifts toward neutral relative to general proteolytic activity; therefore, remarkable kinin production can be expected even at pH > 6. Sap9 optimally cleaved LK at pH 5.5 but, again, the pH optimum of this activity is relatively broad. A number of soluble Saps that exhibit optimal kininogenase activity at a pH as low as 3 (Sap8) and as high as 6 (Sap4) could potentially collaborate with Sap9. Thus, the extensive generation of active kinins from kininogens can potentially occur in most *C. albicans*-infected host niches.

The findings of our current study need to be discussed in the light of previous reports on the actual presence of individual Saps at infection foci. By applying multiple infection models and different methodologies, the available data are both diverse and controversial. Moreover, most studies primarily focus on the expression of Sap-encoding mRNA [50,51] and only occasionally quantify protein products using western blot analysis [52], but these studies do not reveal anything about the *in situ* proteolytic activities of individual Saps. However, the hypothesis that soluble Sap3 alone, or membrane-bound Sap9 with the assistance of several soluble Saps, controls kinins at the sites of candidal infections seems to be relatively insensitive to the discrepancies in the literature because it is difficult to find any studies that definitely exclude the expression of both *SAP3* and *SAP9* from any infection model. This subject has been extensively reviewed [25,35], but only a few representative findings can be cited here. In a study by Albrecht et al. [53], the high, constitutive expression of the *SAP9* gene was consistently detected in both reconstituted human epithelium (RHE) models and human patient samples. Naglik et al. [8,51] reported the common expression of the *SAP2*, *SAP5*, *SAP9* and *SAP10* genes in *C. albicans* isolates obtained from the oral cavity and vagina in human patients. *SAP* gene expression profiles, as well as their order of appearance during infection, differ between oral and vaginal candidiases. For instance, *SAP1*, *SAP3*, and *SAP8* are more commonly expressed in vaginal rather than oral infections in humans [25,35]. In a study on the oral RHE model, *SAP1* and *SAP3* were initially expressed, followed by *SAP6*, and finally *SAP2* and *SAP8* during the late phase of infection [50]; in the vaginal RHE model, the initial expression of the *SAP2*, *SAP10*, and *SAP9* genes was followed by the appearance of the *SAP1*, *SAP4* and *SAP5* transcripts, and finally the expression of *SAP6* and *SAP7* [49]. In both oral and vaginal RHE infected by *sap*-deficient mutants, Sap1–3 were found to play predominant roles in mucosal tissue damage during the initial phase of the infection [49,50]. The results obtained in various animal models are more diverse, but some studies report the expression of all *SAP* genes in murine gastrointestinal infection [54] or the strong, sustained expression of *SAP5* and *SAP9* in murine oropharyngeal candidiasis [55].

## Conclusions

The abilities of most of the 10 *C. albicans* Saps to cleave human kininogen and release kinins, and the available literature on *SAP* gene expression during candidal infections strongly support the hypothesis that kinins are effectively produced at the infection foci during many types of candidiasis and contribute to the inflammatory state of this disease in humans. While small or moderate levels of kinins can be generated by all Saps (except Sap7 and Sap10), the highest level of kinin production depends on the actions of soluble Sap3 or the combined actions of cell wall-bound Sap9 and other soluble Saps such as Sap1, Sap2, Sap4, Sap5, Sap6, and Sap8. The relative contributions of these two mechanisms to potentially generate kinin at infection foci seem to depend on both the type and phase of infection.

## Methods

### Expression and purification of recombinant Saps

The 10 Sap isoenzymes were overproduced in *P. pastoris* GS115 (Invitrogen, Carlsbad, CA) using previously described methods with minor modifications [26]. However, instead of affinity chromatography, final purification consisted of anion-exchange chromatography performed on a Mono Q HR 10/10 column (GE Healthcare, Buckinghamshire, UK) that was equilibrated with 20 mM Tris–HCl (pH 7.5) and eluted using a 0–0.3 M NaCl linear gradient. The proteolytic activity of the purified isozymes was assayed on the BODIPY FL casein substrate (Invitrogen) in 0.1 M buffer that had been adjusted to the optimum pH for each respective Sap (i.e., citrate [pH 3.0–5.0] or phosphate [pH 6.0–7.0]) [26]. Substrate cleavage was monitored using fluorometric measurements at 485-nm excitation and 530-nm emission wavelengths. The protein concentration was determined using the Bradford method [56]. Sap purity was analyzed using SDS-PAGE in the Laemmli system [57], and was determined to be > 95% for each Sap. The identities of all Saps were confirmed using LC-MS/MS after SDS-PAGE and in-gel trypsin digestion [58]. Briefly, the protein bands, which were visualized using colloidal staining with Coomassie Brilliant Blue dye [59], were excised from the gel and washed twice with 50 mM (NH_4_)HCO_3_ in 50% acetonitrile (ACN) (HPLC gradient-grade; Merck, Darmstadt, Germany) at both room temperature and 37°C in order to remove any dye, and then reduced using 10 mM dithiothreitol (DTT) for 60 minutes at 60°C, alkylated with 55 mM iodoacetamide for 45 minutes at room temperature in the dark, washed with 100 mM (NH_4_)HCO_3_, and dehydrated with ACN. After re-swelling on ice in the digestion buffer, which contained 12.5 ng/μL of sequence-grade modified trypsin (Promega, Madison, WI), 50 mM (NH_4_)HCO_3_, and 4 mM CaCl_2_, the gel pieces were incubated overnight at 37°C. Acidic peptides were extracted by adding 50 mM (NH_4_)HCO_3_ and basic peptides with 5% formic acid in 50% ACN. The supernatants were then combined, diluted with water, and subjected to LC-MS/MS analysis (see below).

### HPLC and MS/MS analysis of the Sap-catalyzed cleavage of HK-D4

The synthetic peptide HK-D4 (Lipopharm, Zblewo, Poland), which contains the ISLMKRPPGFSPFRSSRIGEIKEETT sequence (10 μM), was incubated with the individual Saps at a substrate:enzyme molar ratio of 50:1 in 50 mM citrate buffer or 25 mM phosphate buffer at 37°C. The buffer pH and incubation time are the experimental variables, and are further specified in the Results section. The reaction was stopped by mixing the sample (100 μL) with 20 μL of 2 M HCl, and the obtained peptides were separated on a Eurosil Bioselect 300–5 C-18 reversed-phase HPLC column (5 μm, 4 × 250 mm) equipped with a pre-column (both from Knauer, Berlin, Germany). The flow rate was 1 mL/minute, and spectrophotometric detection was performed at 215 nm. Separation was performed using a two-solvent system (solvent A consisted of 0.1% trifluoroacetic acid [TFA] in water; solvent B consisted of 0.08% TFA in 80% ACN), with a linear gradient of 10–30% solvent B for 50 minutes using a Shimadzu LC-10A VP HPLC system (Kyoto, Japan) (two model LC-10 AD VP pumps, SIL-10 AD VP auto injector, SPD-10A VP UV–vis detector, SCL-10A VP system controller and CLASS-VP ver. 5.032 software). For MS/MS identification, the peptide fractions were collected, evaporated to dryness, redissolved in 30% methanol (Merck, Darmstadt, Germany) containing 0.1% formic acid (Sigma-Aldrich), and injected into the ion source of the mass spectrometer (see below for additional details).

### Analysis of Sap-catalyzed LK cleavage

LK (1.5 μM; Sigma-Aldrich) was digested with 0.03 μM Sap in citrate buffer (pH 5.0) for 6 or 24 hours at 37°C. The reaction was stopped using pepstatin A (at a final concentration of 10 μM), and the samples were (1) analyzed for kinin content using a competitive radioreceptor assay, (2) acidified with HCl (to a final concentration of 0.33 M) and subjected to LC-MS/MS analysis (see below), or (3) acidified with HCl and subjected to HPLC separation using Shimadzu chromatograph (as specified above) on the Luna C18(2) column (5 μm, 4.6 × 250 mm) (Phenomenex, Torrance, CA) in the binary gradient formed between 0.1% TFA in water (solvent A) and 0.08% TFA in 80% ACN (solvent B). Separation was performed according to the following time program: 20–29% solvent B in 45 minutes, and 29–100% solvent B in 10 minutes. The fractions were separately collected at the retention times that corresponded to the bradykinin, Met-Lys-bradykinin, and des-Arg^1^-bradykinin standards, evaporated to dryness, and redissolved in the assay buffer provided with the ELISA kit in order to quantitatively determine the kinin concentration (see below).

### LC-MS/MS

The equipment used for the LC-MS/MS analysis consisted of an HCTultra ion-trap (IT) mass spectrometer equipped with an electrospray ionization (ESI) ion source and electron transfer dissociation (ETD II) fragmentation module (Bruker, Bremen, Germany), which were coupled to the ultrahigh-performance liquid chromatography (UHPLC) Dionex Ultimate 3000 system (Thermo Scientific, Waltham, MA). An Accucore C-18 column (2.6 μm, 2.1 × 100 mm; Thermo Scientific) was used to separate peptides in a binary gradient of 10-25% solvent B in 38 minutes, at a flow rate of 0.2 mL/minute (solvent A consisted of 0.1% spectrophotometric-grade formic acid [Sigma-Aldrich] in LC-MS grade water; solvent B consisted of 0.1% formic acid in 80% ACN). The LC separation step was omitted for some analyses, and the samples were directly injected using a syringe pump (KD Scientific, Holliston, MA) at a flow rate of 180 μL/hour. All mass spectrometric measurements were performed in positive ion mode with a capillary voltage of 3.5 kV, a nebulizing gas (nitrogen) pressure of 10 psi, drying gas (nitrogen) flow of 5 L/minute, and dry gas temperature of 300°C. Spectra were acquired in MS/MS mode in the range of 100–3000 m/z with ETD ion fragmentation. Tandem MS data analysis was performed using DataAnalysis™ 4.0 and Biotools™ 3.2 software (Bruker, Germany), and identification was performed by searching the Swiss-Prot database using in-house Mascot server (ver. 2.3.0; Matrix Science, London, UK).

### Competitive radioreceptor assay for evaluating kinins

HEK293 cells that stably overexpressed the kinin B2 receptors were obtained using the Flp-In T-Rex-System (Invitrogen), as previously described [60]. Cells were cultured in Dulbecco’s modified Eagle’s medium (DMEM) with a high-glucose concentration, 2 mM glutamine, and sodium pyruvate (CytoGen GmbH, Sinn, Germany) and supplemented with 10% fetal bovine serum (FBS) (Lonza, Switzerland), 0.1 mg/mL penicillin/streptomycin (CytoGen GmbH), and 250 ng/mL amphotericin B (CytoGen GmbH). Medium was replaced every 3–4 days, and cells were routinely subcultured with trypsin-EDTA (CytoGen GmbH) for detachment and transfer. For all experiments, cells (5 × 10^4^) were transferred to the wells of 96-well plates that had been pretreated with 0.01% polylysine (Sigma-Aldrich) in phosphate-buffered saline (PBS) (CytoGen GmbH) and cultured to 80–100% confluence.

The radioreceptor assay was performed according to a previously described method [28]. Before all experiments, the cell monolayers were washed three times with cold PBS and incubated for half an hour in the equilibration buffer (40 mM PIPES, 100 mM NaCl, 5 mM KCl, 0.1% glucose, 0.05% bovine serum albumin [BSA], 2 mM CaCl_2_, and 1 mM Mg_2_Cl_2_ [pH 7.4]) that had been supplemented with peptidase inhibitors (80 μM 1,10-phenantroline [Sigma-Aldrich], 100 μM captopril [Fluka, Buchs, Switzerland], and 2 mM bacitracin [Sigma-Aldrich]). After removing the buffer, the cells were incubated on ice with a mixture of 2 nM [^3^H]-bradykinin (Perkin-Elmer, Waltham, MA) and bradykinin, Met-Lys-bradykinin, or des-Arg^1^-bradykinin. The concentrations of the unlabelled peptides in the mixtures varied between 0.05 nM–50 μM. After 90 minutes, the cells were washed four times with PBS and incubated with 200 μL of 0.2 M acetate buffer with 0.5 M NaCl (pH 2.7). The supernatants with the dissociated [^3^H]-labeled tracer peptides were transferred to vials containing 2.5 mL of Ultima Gold™ scintillation fluid (Perkin-Elmer), and radioactivity was measured using a beta counter (Wallace 1412; LKB, Uppsala, Sweden).

### Kinin determination using ELISA

The kinin concentration was determined using the Bradykinin-EIA kit (Peninsula Laboratories/Bachem, San Carlos, CA) according to the manufacturer’s instructions. In this assay, the kinin in each sample competes with a fixed concentration of biotinylated bradykinin to bind with the antiserum that is immobilized to the microplate well. The concentration of bound biotinylated tracer was determined using streptavidin-conjugated horseradish peroxidase/3,3^′^,5,5^′^-tetramethylbenzidine (SA-HRP/TMB), and the kinin content of the sample was estimated from the calibration curve prepared for the kinin concentration range (0–5 nM). Because the antibody used in this assay was specific to the 5 amino acid C-terminal sequence of bradykinin, identical calibration plots were obtained using bradykinin, Met-Lys-bradykinin, and des-Arg^1^-bradykinin.

## Abbreviations

ACN: Acetonitrile
BSA: Bovine serum albumin
DMEM: Dulbecco’s modified Eagle’s medium
DTT: Dithiothreitol
ELISA: Enzyme-linked immunosorbent assay
ESI: Electrospray ionization
ETD: Electron transfer dissociation
FBS: Fetal bovine serum
HEK: Human embryonic kidney
HK-D4: ISLMKRPPGFSPFRSSRIGEIKEET peptide
HK: High-molecular-mass kininogen
HPLC: High-performance liquid chromatography
IT: Ion trap
LC-MS/MS: Liquid chromatography-coupled tandem mass spectrometry
LK: Low-molecular-mass kininogen
MS: Mass spectrometry
MS/MS: Tandem mass spectrometry
PBS: Phosphate-buffered saline
RHE: Reconstituted human epithelium
Sap: Secreted aspartic protease
SDS-PAGE: Sodium dodecyl sulphate polyacrylamide gel electrophoresis
TFA: Trifluoroacetic acid
UHPLC: Ultrahigh-pressure liquid chromatography
